# Dynamic Responses of Ammonia-Oxidizing Archaea and Bacteria Populations to Organic Material Amendments Affect Soil Nitrification and Nitrogen Use Efficiency

**DOI:** 10.3389/fmicb.2022.911799

**Published:** 2022-05-12

**Authors:** Jie Zheng, Liang Tao, Francisco Dini-Andreote, Lu Luan, Peijun Kong, Jingrong Xue, Guofan Zhu, Qinsong Xu, Yuji Jiang

**Affiliations:** ^1^State Key Laboratory of Soil and Sustainable Agriculture, Institute of Soil Science, Chinese Academy of Sciences, Nanjing, China; ^2^University of Chinese Academy of Sciences, Beijing, China; ^3^Guangdong Key Laboratory of Integrated Agroenvironmental Pollution Control and Management, Institute of Eco-environmental and Soil Sciences, Guangdong Academy of Sciences, Guangzhou, China; ^4^Department of Plant Science and Huck Institutes of the Life Sciences, The Pennsylvania State University, University Park, PA, United States; ^5^College of Life Science, Nanjing Normal University, Nanjing, China

**Keywords:** ammonia oxidizers, competitive interaction, keystone taxa, potential nitrification activity, plant productivity, straw application

## Abstract

Organic material amendments have been proposed as an effective strategy to promote soil health by enhancing soil fertility and promoting nitrogen (N) cycling and N use efficiency (NUE). Thus, it is important to investigate the extent to which the structure and function of ammonia-oxidizing archaea (AOA) and bacteria (AOB) differentially respond to the organic material amendments in field settings. Here, we conducted a 9-year field experiment to track the responses of AOA and AOB populations to the organic material amendments and measured the potential nitrification activity (PNA), plant productivity, and NUE in the plant rhizosphere interface. Our results revealed that the organic material amendments significantly enhanced the abundance and diversity of AOA and AOB populations. Further, significant differences were observed in the composition and co-occurrence network of AOA and AOB. A higher occurrence of potential competitive interactions between taxa and enumerated potential keystone taxa was observed in the AOA-AOB network. Moreover, we found that AOA was more important than AOB for PNA under the organic material amendments. Structural equation modeling suggested that the diversity of AOA and AOB populations induced by the potential competitive interactions with keystone taxa dynamically accelerated the rate of PNA, and positively affected plant productivity and NUE under the organic material amendments. Collectively, our study offers new insights into the ecology and functioning of ammonia oxidizers and highlights the positive effects of organic material amendments on nitrogen cycling dynamics.

## Introduction

Nitrogen (N) is the key limiting element to control the growth of plants in terrestrial ecosystems. The application of inorganic N fertilizers provides continuous support to crop systems, but its increased use is neither energy-efficient nor environmentally desirable (Canfield et al., 2010; Springmann et al., 2018). For instance, it has been estimated that more than half of N used in agricultural settings are susceptible to being lost, thereby reducing N cycling and N use efficiency (NUE; Bindraban et al., 2015). Straw can function as a form of organic material that integrates N fertilization with organic carbon and available nutrients (Chen et al., 2017). For instance, straw in combination with manure has been reported to properly adjust the C/N ratio (Qiao et al., 2015), and straw biochar was shown to increase soil pH and N availability (Chen et al., 2019; Wang et al., 2020). As such, organic material amendments can be seen as an effective practice to improve crop productivity by increasing soil fertility and N cycling rate through nitrification (Wang et al., 2015).

Ammonia oxidation is the first and rate-limiting step of soil nitrification (Leininger et al., 2006). The conversion of ammonia (NH_3_) to nitrite (NO_2_^−^) is performed by a diverse group of ammonia oxidizers, ammonia-oxidizing archaea (AOA), and bacteria (AOB), respectively (Martens-Habbena et al., 2009). Insights into the dynamics of these groups and their niche preferences have pointed to an overall prevalence of AOA over AOB in acidic soils, while AOB operates under high NH_3_ concentrations and prefers neutral to low alkaline soils (Prosser and Nicol, 2012; Zhang et al., 2012). The abundance and diversity of AOA and AOB populations and potential nitrification activity (PNA) have been shown to change with soil environmental factors (pH and N availability) and field management regimes (organic material amendments; Jiang et al., 2015; Zhang et al., 2019). For example, the long-term straw combined with manure application was found to increase the abundance and diversity of AOA and AOB populations mostly due to the high level of available N (Li et al., 2015). The application of straw or straw biochar was reported to significantly stimulate AOB abundance and diversity, while neutral to negative effects were observed on AOA abundance (He et al., 2016; Lin et al., 2020). However, we still lack a comprehensive understanding of organic material amendments regulating the community of ammonia oxidizers as does the effect on soil nitrification.

Co-occurrence network analysis has been used to provide insights into potential biological interactions between taxa, and to enumerate and infer potential keystone taxa (Fuhrman, 2009). Keystone taxa likely play an important role in determining ecological interactions between taxa by affecting network stability and modularity (Herren and McMahon, 2018). It has been suggested that specific keystone taxa in the AOA and AOB populations are tightly linked to soil PNA when compared to other ammonia oxidizers in the network (Yang et al., 2021). In this sense, ammonia oxidizers may exhibit cooperation with keystone taxa to enhance AOB diversity and PNA with pig manure application (Jiang et al., 2015). Therefore, it is reasonable to hypothesize that organic material amendments can exert stimulatory effects on the ammonia oxidizer communities and PNA by regulating potential keystone taxa. So far, the mechanism of potential keystone taxa in the AOA and AOB populations mediating soil nitrification and NUE is an open question.

Here, we studied the dynamic responses of AOA and AOB populations, and soil nitrification to the organic material amendments. We conducted a 9-year field experiment subjected to five fertilization treatments in low-fertile red soil. In particular, we addressed the following questions: (1) what are the effects of different organic material amendments on the abundance, diversity, composition, and co-occurrence patterns of AOA and AOB populations? (2) Whether and to what extent does the dynamic of AOA and AOB populations induced by keystone taxa affect soil PNA, plant productivity, and NUE? We hypothesized that organic material amendments significantly enhanced the abundance and diversity of AOA and AOB populations, and altered the composition of ammonia oxidizers. We further expected that the network module with AOA and AOB keystone taxa would be significantly associated with PNA, plant productivity, and NUE.

## Materials and Methods

### Description of the Field Experiment

The long-term fertilization experiment was established at the Red Soil Ecological Experimental Station of the Chinese Academy of Sciences (28°15′N, 116°55′E), Yingtan, Jiangxi. The climate condition in the area is classified as warm and monsoon with a mean annual temperature and precipitation of 17.6°C and 1,795 mm. The soil was classified as Ferric Acrisol according to the FAO classification system. This experiment started in 2010 and was maintained for a period of 9 years. The complete randomized design (20 m × 5 m) includes five treatments with three replicates, as follows: no fertilizer (CK); NPK fertilizer (N), NPK fertilizer and straw (NS); NPK fertilizer and straw combined with pig manure (NSM); and NPK fertilizer and straw biochar (NB). The straw application consisted of naturally air-dried maize straw fragmented into short rod-like particles (2–5 mm diameter × 1–5 cm length). The air-dried maize straw was converted to biochar by pyrolyzed at 450°C for 48 h. The concentrations of straw, pig manure, and biochar were calculated according to the annual carbon input of 1,000 kg ha^−1^. The application of straw and pig manure was applied according to a carbon ratio of 9:1 on a dry matter basis. The NPK fertilizers were applied at 150 kg N ha^−1^, 75 kg P_2_O_5_ ha^−1^, and 60 kg K_2_O ha^−1^. The total N amount applied to the CK, N, NS, NSM, and NB treatments were 0, 150, 166, 172.35, and 185.57 kg ha^−1^, respectively. The field was annually used for corn production as a monoculture (cultivar Suyu No. 24) from April to July. Straw, pig manure, and biochar were applied in the field together with NPK, followed by plowing and even mixing before seeding. No additional practices were carried out in the field, except manual weed control.

### Soil Sampling and Determination of Chemical Properties

Soil samples were collected after the harvest in late July of 2019. At each plot, 10 plants were randomly selected to collect rhizosphere samples from roots at a depth of 0–15 cm. After shaking off the loosely adhering soil to the 10 plant roots, the tightly adhering rhizosphere soil was collected and finally mixed as a composite sample. Rhizosphere samples were placed on ice and immediately transported to the laboratory (<24 h) for chemical and microbial analyses.

Soil pH was determined using a glass electrode in a 1:2.5 soil: water solution (*w*/*v*). Soil organic carbon (SOC) was determined using the potassium dichromate method (Nelson and Sommers, 1996), and total nitrogen (TN) was measured by the micro-Kjeldahl method (Bremner, 1996). NO_3_^−^-N and NH_4_^+^-N were extracted with 2 M KCl and determined on a continuous flow analyzer (Skalar, Breda, Netherlands). Soil water content was calculated as the ratio of the mass of water lost by dying to the mass of oven-dried soil.

### Potential Nitrification Activity, Plant Productivity, and N Use Efficiency

Potential nitrification activities of AOA and AOB populations were determined following a previously modified protocol (Taylor et al., 2013). In brief, PNA with 1-octyne (4 μM) was attributed to AOA populations, and the contribution of AOB populations was the difference between PNA without 1-octyne and PNA with 1-octyne. A total of 5 g of fresh soil (equivalent dry mass) was added to 50 ml flasks containing 2 mM (NH_4_)_2_SO_4_ and 10 mM sodium chlorate (NaClO_3_, an inhibitor of nitrite oxidation) solution and shook at 150 rpm at 25°C (Ke et al., 2013). A volume of 2 ml aliquots of the suspension was sampled after 0, 8, 24, 30, 48, 60, and 72 h, respectively. The filtrate was used to determine the concentration of NO_2_^−^ in the slurries to further determine the PNA. Straw biomass and grain yield of maize from each plot were measured after sun drying, and the dry weight of straw and grain was expressed as kilogram per hectare. The total N concentrations of straw and grain were determined using the semi-micro-Kjeldahl method. NUE was calculated as the difference between N uptake in the treatment with N fertilizer and the N uptake in the treatment without N fertilizer, divided by the N application rate (Su et al., 2014).

### Soil DNA Extraction and Illumina Sequencing

Total DNA was extracted from 0.5 g of rhizosphere soil using the Mo-Bio Power Soil DNA Extraction Kit (MoBio, California, United States), according to the manufacturer’s protocol. DNA quality and concentration were determined using a Nanodrop 2000C Spectrophotometer. The archaeal and bacterial *amoA* genes were amplified using the primer pairs Arch-*amoA* 26F/Arch-*amoA* 417R (Park et al., 2008) and *amoA*-1F/*amoA*-2R (Rotthauwe et al., 1997), respectively. The 20 μl PCR mixture contained 10 μl 2 × SYBR® Premix, 0.5 μM of each primer, and 1 μl DNA template (1–10 ng). The PCR protocol was as follows: an initial pre-denaturation at 94°C for 2 min; 30 cycles of 30 s at 94°C, 30 s at 55°C, and 30 s at 72°C; and a final extension at 72°C for 10 min. The obtained amplicons were purified in equimolar concentrations in a single tube, and then they were subjected to library preparation, cluster generation, and paired-end 2 × 300 bp sequencing using the Illumina MiSeq platform.

### Quantitative PCR of *amoA* Gene of AOA and AOB

Quantitative PCR (qPCR) was performed to assess the copy number of AOA and AOB *amoA* genes. The qPCR was performed in 20 μl reaction mixtures containing the following components: 10 μl [2 × SYBR® Premix Ex Taq (TaKaRa, Dalian, China)], 0.5 μM of each primer described above, 1 μl DNA template (1–10 ng). No-template controls were included with each qPCR run. The qPCR products were cloned into a plasmid using the procedures reported by Okano et al. (2004). Standard curves were constructed using a dilution series (10^2^–10^8^ copies) of plasmids DNA for quantifying gene copy numbers. All qPCR assays were run starting with the initial denaturation step at 95°C for 3 min, followed by 40 cycles (with plate-reading) of 30 s at 95°C and 45 s at 60°C, then with a final melt curve step from 72 to 95°C. The qPCR was run in triplicate, and amplification efficiencies of >97% were obtained with *R*^2^ values for the standard curves were higher than 0.99.

### Sequence Analysis

Raw sequences were processed using Quantitative Insights into Microbial Ecology Version 2 (QIIME2; Bolyen et al., 2019). Sequences that fully matched the barcodes were selected and subjected to quality trimming, demultiplexing, clustering, and taxonomic assignments. The sequences of low quality (quality score < 25 and length < 250 bp) were excluded and the remaining sequences were further filtered using chimera detection in UCHIME with a reference data set (Edgar et al., 2011). After that, the sequence reads from each sample were screened for frame shifts using the FrameBot tool of RDP’s FunGene Pipeline. The obtained quality-screened sequences were then clustered into operational taxonomic units (OTUs) using a Cluster Database at High Identity with Tolerance (CD-HIT) at 90% of sequence identity after sequences were subjected to a similarity search (Li and Godzik, 2006). The OTUs were taxonomically categorized using neighbor-joining phylogenetic trees constructed from representative sequences with the longest length of the *amoA* genes. AOA *amoA* representatives were matched with an existing high-quality *amoA* database (Pester et al., 2012), while AOB *amoA* representatives were matched with GeneBank reference sequences. The taxonomy-derived reference sequences were combined with the Kimura 2-parameter distance (MEGA version 7.0), and the bootstrap values were calculated using 1,000 repetitions (Kumar et al., 2016).

### Statistical Analysis

One-way analysis of variance (ANOVA) and Spearman correlation analysis was performed using Tukey’s HSD in SPSS 24.0 (SPSS, Chicago, IL, United States). Non-metric multidimensional scaling (NMDS) analysis was conducted to determine the community composition based on the Bray–Curtis dissimilarity between samples using the R package vegan, and the compositional similarity was calculated as 1 minus the Bray–Curtis dissimilarity (Oksanen et al., 2018). Network analysis was performed by calculating multiple correlations between taxa using CoNet (Faust et al., 2012). Only OTUs detected in more than three-fourths of the samples at the same depth were kept for the analysis. A valid co-occurrence was considered statistically robust when the correlation coefficient (*r*) was >0.7 (positive) or <−0.7 (negative) and the *p*-value was <0.01. *p*-Values were adjusted using the Benjamini–Hochberg procedure to avoid false positives (Benjamini and Hochberg, 1995). Network visualization was conducted using the Gephi software (Bastian et al., 2009). Nodes indicated individual AOA and AOB taxa (OTUs), and network edges indicated pairwise correlations between nodes (Weiss et al., 2016). Network modules were defined as clusters of closely connected nodes (groups of co-occurring microbes). The AOA and AOB networks were searched to identify highly associated nodes using the Molecular Complex Detection (MCODE) in the Cytoscape platform. The topological role of each node was estimated by two topological parameters, such as the within-module connectivity *Z* and the among-module connectivity *P* (Olesen et al., 2007). The nodes with *Z* > 2.5 and *p* < 0.62 were sorted into module hubs and classified as potential keystone taxa. The first principal component of the modules (module eigengenes) was calculated in the standardized module expression data for the co-occurrence networks (Langfelder and Horvath, 2007). Natural connectivity was applied to test the network’s structural robustness (Wu et al., 2010). We conducted random forest modeling to quantitatively estimate the contribution of predictors to PNA, plant productivity, and NUE. Modeling was constructed using the “randomForest” package (Liaw and Wiener, 2002), and the significance of the corresponding model was measured using the “A3R” and “rfPermute” packages, respectively (Fortmannroe, 2015; Archer, 2020). Structural equation modeling (SEM) was applied to assess potential direct and indirect effects of soil properties and AOA and AOB populations, affecting plant productivity, and NUE. The framework and rationale of the priori SEM shown in Supplementary Figure S1 are based on our hypotheses. These analyses were carried out using the AMOS 20.0 software with the maximum likelihood estimation. The chi-square value (*p* > 0.05), goodness-of-fit index, root mean square error of approximation, and Akaike information criterion were used to examine the fit of SEM model (Hooper et al., 2008).

## Results

### Soil Chemical Properties, Plant Productivity, and N Use Efficiency

**Figure 1:**
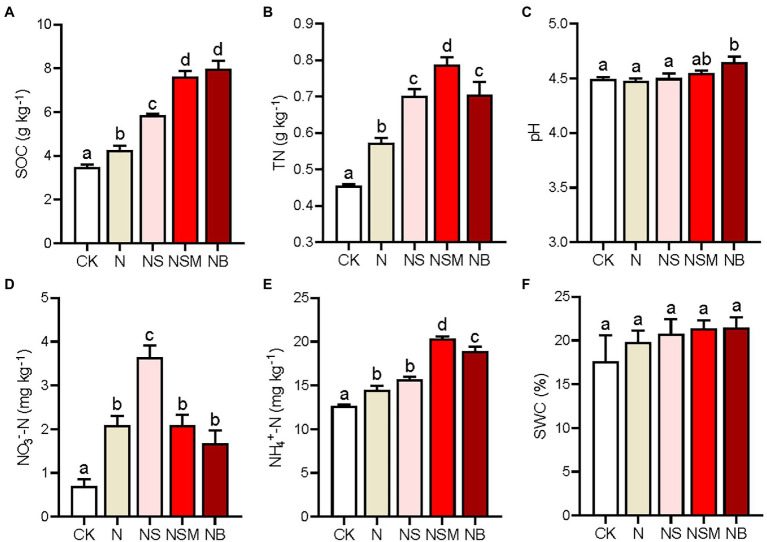
Chemical properties of the rhizosphere soils under the five fertilization treatments, including soil organic carbon (SOC, **A**), total nitrogen (TN, **B**), pH **(C)**, nitrate nitrogen (NO_3_^−^-N, **D**), ammonia nitrogen (NH_4_^+^-N, **E**), and soil water content (SWC, **F**). Different lowercase letters indicate significant differences based on Tukey’s HSD test (*p* < 0.05). CK, no fertilizer; N, NPK fertilizer; NS, NPK fertilizer and straw; NSM, NPK fertilizer and straw combined with pig manure; NB, NPK fertilizer and straw biochar.

The results of one-way ANOVA analysis showed that the three organic material amendments (NS, NSM, and NB) significantly increased SOC and TN compared to the CK and N treatments (Figures 1A,B; *p* < 0.05). Soil TN was the highest under the NSM treatment, and SOC was the highest under the NB treatment. Soil pH was significantly higher under the NB treatment than under the CK, N, and NS treatments (Figure 1C; *p* < 0.05). NO_3_^−^-N and NH_4_^+^-N contents ranged from 0.7 to 3.64 mg kg^−1^ and from 12.62 to 20.35 mg kg^−1^, respectively. Compared to the N treatment, NO_3_^−^-N was significantly increased under the NS treatment, while NH_4_^+^-N was substantially improved under the NSM and NB treatments (Figures 1D,E; *p* < 0.05). However, no significant difference in soil water content was observed across the five treatments (Figure 1F; *p* > 0.05).

**Figure 2:**
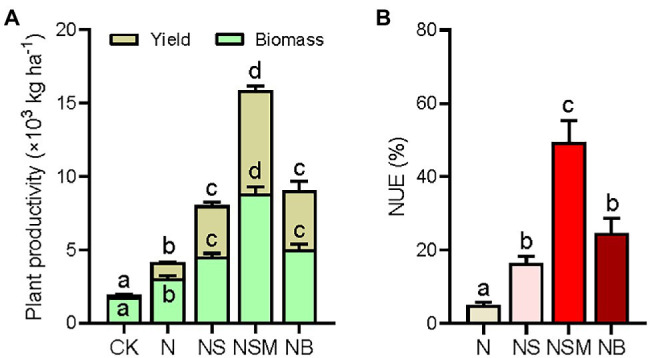
Plant productivity (grain yield and straw biomass, **A**) and nitrogen use efficiency (NUE, **B**) under the five fertilization treatments. Different lowercase letters indicate significant differences based on Tukey’s HSD test (*p* < 0.05). CK, no fertilizer; N, NPK fertilizer; NS, NPK fertilizer and straw; NSM, NPK fertilizer and straw combined with pig manure; NB, NPK fertilizer and straw biochar.

Plant productivity was significantly (*p* < 0.05) higher under the three organic material amendments than under the CK and N treatments, including straw biomass and grain yield of maize (Figure 2A). Straw biomass and grain yield of maize were the highest under the NSM treatment, increasing by 2.9- and 6.7-fold compared to the N treatment, respectively. NUE was found to follow a similar trend as plant productivity and increased by 11.3, 43.2, and 19.6% under the NS, NSM, and NB treatments relative to the N treatment (Figure 2B).

### Abundance, Diversity, Composition, and Nitrification of AOA and AOB Populations

The abundance, diversity, and composition of AOA and AOB populations were analyzed using qPCR and Illumina sequencing targeting the *amoA* gene. The abundance and diversity of *amoA* gene in AOA and AOB populations responded significantly different to three organic material amendments (Table 1; *p* < 0.05). The AOA and AOB abundances across the five treatments ranged from 0.31 × 10^5^ to 7.16 × 10^6^ copies g^−1^ dry soil and from 0.26 × 10^4^ to 4.93 × 10^4^ copies g^−1^ dry soil, respectively. The AOA and AOB abundances under the NS, NSM, and NB treatments increased by 4.88-, 9.64-, and 1.93-fold and by 4.36-, 3.93-, and 5.42-fold relative to the N treatment, respectively (Table 1; *p* < 0.01). The NSM treatment had a significantly (*p* < 0.05) higher AOA/AOB ratio than the N treatment, while the NB treatment followed an opposite trend (Table 1). The AOA and AOB diversity indices estimated by Shannon index and Chao1 richness were significantly higher under the NS, NSM, and NB treatments compared to the CK and N treatments (Table 1; *p* < 0.05).

**Table 1:** The abundance, diversity, and potential nitrification activity (PNA) of AOA and AOB populations under the five fertilization treatments.^a^

	Ammonia oxidizers	CK	N	NS	NSM	NB
Abundance^b^	AOA (×10^5^ copies g^−1^ dry soil)	0.31 ± 0.03a	7.42 ± 0.67b	36.23 ± 5.74c	71.57 ± 6.31d	14.30 ± 1.19bc
	AOB (×10^4^ copies g^−1^ dry soil)	0.26 ± 0.04a	0.91 ± 0.05b	3.97 ± 0.28c	3.58 ± 0.09c	4.93 ± 0.38d
	AOA/AOB ratios^b^	12.99 ± 3.55a	82.86 ± 12.07c	94.37 ± 22.12cd	200.12 ± 19.42d	29.57 ± 4.39b
Shannon index^b^	AOA	1.04 ± 0.04a	1.27 ± 0.09b	1.97 ± 0.05c	1.88 ± 0.08c	2.37 ± 0.12d
	AOB	100.75 ± 5.80a	116.41 ± 7.96ab	178.09 ± 6.98c	170.12 ± 4.08c	138.44 ± 9.43b
Chao1 richness^b^	AOA	1.63 ± 0.09b	1.23 ± 0.06a	1.97 ± 0.11d	1.72 ± 0.08bc	1.87 ± 0.05c
	AOB	94.70 ± 3.55b	71.04 ± 8.3a	252.99 ± 8.99d	282.90 ± 7.45e	132.71 ± 13.74c
PNA^b^	PNA_AOA_ (μg NO_2_^−^-N dry soil h^−1^)	0.015 ± 0.001a	0.024 ± 0.004ab	0.055 ± 0.002c	0.058 ± 0.004c	0.028 ± 0.005b
	PNA_AOB_ (μg NO_2_^−^-N dry soil h^−1^)	0.007 ± 0.001a	0.011 ± 0.001a	0.026 ± 0.002b	0.033 ± 0.006b	0.027 ± 0.001b

a *Values are the means (*n* = 3) ± the standard error of the mean. Values in the same column followed by a lowercase letter indicate significant differences revealed by Tukey’s HSD test (*p* < 0.05). CK, no fertilizer; N, NPK fertilizer; NS, NPK fertilizer and straw; NSM, NPK fertilizer and straw combined with pig manure; NB, NPK fertilizer and straw biochar*.

b *The abundance of ammonia-oxidizing archaea (AOA) and bacteria (AOB) are indicated by *amoA* gene copies numbers. AOA/AOB ratios, the ratios of AOA to AOB. The diversity of AOA and AOB are estimated by Shannon index and Chao1 richness. PNA_AOA_, the potential nitrification activity of AOA; PNA_AOB_, the potential nitrification activity of AOB*.

**Figure 3:**
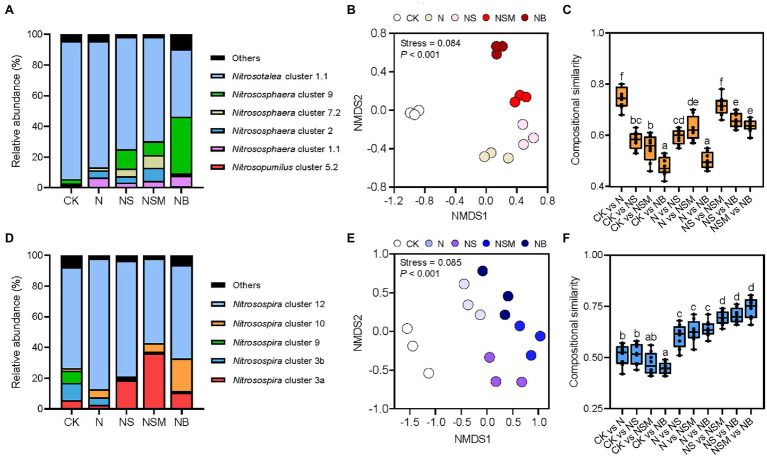
The ammonia oxidizers under the five fertilization treatments. Taxonomic information of AOA **(A)** and AOB **(D)** populations are based on the *amoA* gene sequences. Non-metric multidimensional scaling (NMDS) analysis of AOA **(B)** and AOB **(E)** population structures based on the *amoA* gene. The pairwise compositional similarity of AOA **(C)** and AOB **(F)** population structures across five treatments. Different lowercase letters indicate significant differences based on Tukey’s HSD test (*p* < 0.05). CK, no fertilizer; N, NPK fertilizer; NS, NPK fertilizer and straw; NSM, NPK fertilizer and straw combined with pig manure; NB, NPK fertilizer and straw biochar.

Organic material amendments were found to have pronounced (*p* < 0.001) effects on the variations in AOA and AOB composition, as determined using non-metric multidimensional scaling analysis (Figures 3B,E). The compositional similarities of AOA and AOB populations among the three organic material amendments were significantly (*p* < 0.05) higher than those between the CK and N treatments (Figures 3C,F). The AOA populations were mostly composed of *Nitrosotalea* (70.8%) and *Nitrososphaera* (25.1%; Figure 3A), which were further divided into *Nitrosotalea* cluster 1.1 (70.8%), and *Nitrososphaera* clusters 1.1 (4.5%), 2 (3.9%), 7.2 (3.6%), and 9 (12.3%). The relative abundance of *Nitrosotalea* cluster 1.1 was significantly (*p* < 0.05) higher under the CK and N treatments, while *Nitrososphaera* clusters 2 and 9 were significantly (*p* < 0.05) higher under the NS, NSM, and NB treatments. The AOB populations mostly consisted of the dominant genus *Nitrosospira* (93.7%), which was affiliated with the *Nitrosospira* clusters 3a (19.2%), 3b (3.9%), 9 (1.9%), 10 (6.8%), and 12 (61.9%; Figure 3D). The relative abundances of AOB *Nitrosospira* clusters 12 and 3b were significantly (*p* < 0.01) higher under the CK and N treatment, respectively, whereas that of *Nitrosospira* cluster 3a was significantly (*p* < 0.01) higher under the NS, NSM, and NB treatments compared with N treatment.

The AOA-specific PNA (PNA_AOA_) was significantly (*p* < 0.05) higher under the NS and NSM treatments relative to the CK, N, and NB treatments (Table 1). The AOB-specific PNA (PNA_AOB_) was significantly (*p* < 0.05) higher under the three organic material amendments compared to the CK and N treatments (Table 1). Correlation analysis indicated that the relative abundances of *Nitrososphaera* clusters 2 and 9 in the AOA population and *Nitrosospira* cluster 3a in AOB population were significantly positively associated with SOC, TN, and NH_4_^+^-N, as well as with PNA_total_, straw biomass, grain yield, and NUE (*r* = 0.44–0.86, *p* < 0.05; Supplementary Figure S2). However, the relative abundances of *Nitrosospira* cluster 3b and 9 were negatively associated with SOC, TN, NO_3_^−^-N, and NH_4_^+^-N, as well as with PNA_total_, straw biomass, grain yield (*r* = −0.56 to −0.93, *p* < 0.05).

### Co-occurrence Patterns of AOA and AOB Without and With Organic Material Amendments

**Figure 4:**
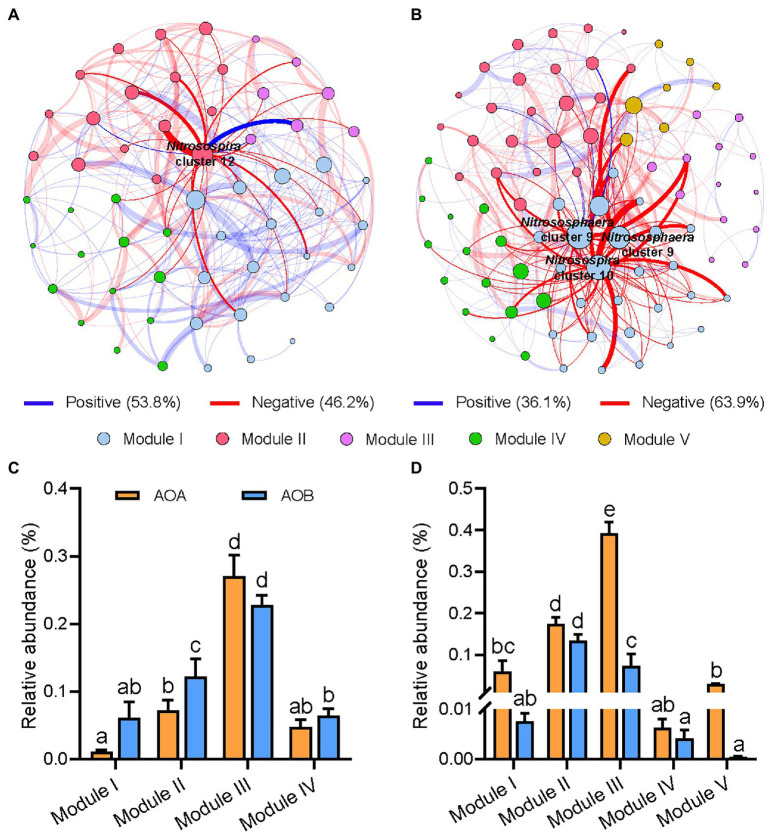
Co-occurrence networks of AOA and AOB populations based on treatments without (OM−; **A**, CK and N) and with organic material amendments (OM+; **B**, NS, NSM, and NB). Relative abundances of AOA and AOB in OM− **(C)** and OM+ **(D)** of network’s module eigengenes. The modules I−V represent the five clusters of closely interconnected nodes. Each node represents an OTU and its size is proportional to the number of connections (degree). The thickness of each connection between two nodes (edge) is proportional to the value of Spearman’s correlation coefficients. Blue and red edges indicate positive and negative correlations, respectively. Different lowercase letters indicate significant differences based on Tukey’s HSD test (*p* < 0.05).

**Figure 5:**
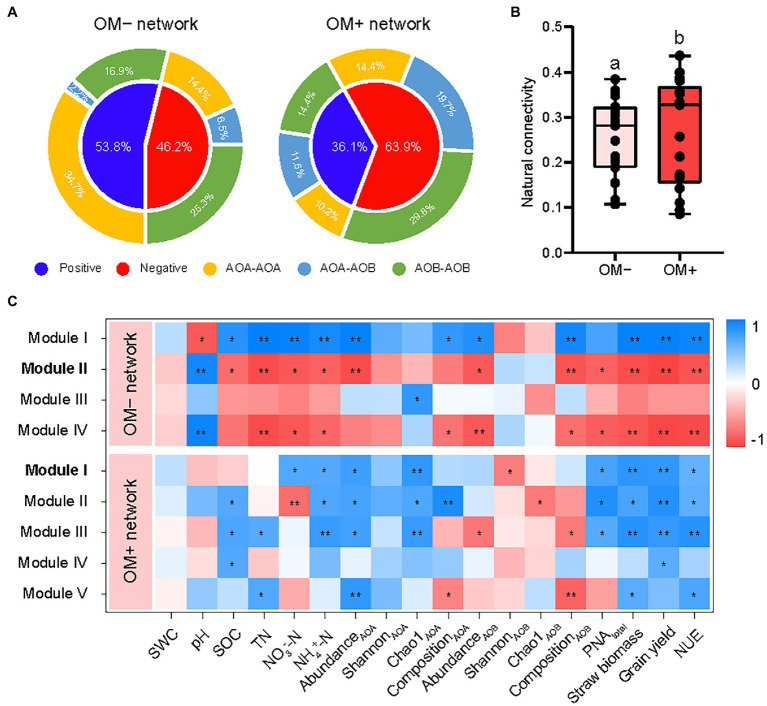
Values of correlation types **(A)** between edges in the networks without (OM−) and with (OM+) organic material amendments. The co-occurrence network robustness **(B)** in OM− and OM+ is indicated by the natural connectivity. Correlation coefficients **(C)** between module eigengenes, soil properties, AOA and AOB populations, PNA_total_, plant productivity (grain yield and straw biomass), and nitrogen use efficiency (NUE). Different lowercase letters indicate significant differences based on Tukey’s HSD test (*p* < 0.05). The modules with keystone taxa in the OM− and OM+ networks are in bold. SWC, soil water content; SOC, soil organic carbon; TN, total nitrogen; NO_3_^−^-N, nitrate nitrogen; NH_4_^+^-N, ammonia nitrogen; PNA_total_, the sum of potential nitrification activity of AOA and AOB. ^**^p < 0.01; ^*^p < 0.05.

Distinct topological characteristics were observed in networks built based on treatments with (OM+) and without (OM−) organic material amendments. The total number of nodes and edges, and the percentage of negative correlations (PNC) in the OM+ were higher compared to those in the OM− (Supplementary Table S1; Figure 4). The number of negative correlations including AOA-AOB and AOB-AOB in the OM+ was higher compared to the OM− (Figure 5A). The values of natural connectivity were significantly (*p* < 0.05) higher in the OM+ than in the OM− (Figure 5B). Based on the topological characteristics, the AOB *Nitrosospira* cluster 12 (4.95%) in the OM−, and the AOA *Nitrososphaera* cluster 9 (0.18 and 2.27%) and AOB *Nitrosospira* cluster 10 (0.01%) in the OM+ were identified as module hubs (Table 2; Figures 4A,B). The *Nitrosospira* cluster 12 was mainly negatively associated with connected members in module II of the OM−, and its relative abundance was negatively related to the AOB Chao1 richness (*r* = −0.57, *p* < 0.05), but positively related to PNA_total_ (*r* = 0.48, *p* < 0.05; Table 2). The genera *Nitrososphaera* cluster 9 and *Nitrosospira* cluster 10 were of intensely negative relevance for the linked nodes in module I of the OM+, but their relative abundances displayed strongly positive relationships with Chao1 richness of AOA and AOB, and PNA_total_ (*r* = 0.52–0.84, *p* < 0.05; Table 2). The relative abundance of AOA nodes in all modules of the OM+ was higher than that of AOB, as well as structuring an exclusive module III in the OM− (Figures 4C,D). In the OM−, module I was positively correlated with SOC, TN, NO_3_^−^-N, NH_4_^+^-N, the abundance and composition of AOA and AOB populations, plant productivity, and NUE (*r* = 0.81–0.99, *p* < 0.05), whereas the modules II and IV exhibited negative correlations (*r* = −0.7 to −0.99, *p* < 0.05; Figure 5C). In the OM+, the modules I, II, and III were positively correlated with NH_4_^+^-N, the abundance and Chao1 richness of AOA population, PNA_total_, plant productivity, and NUE (*r* = 0.61–0.86, *p* < 0.05; Figure 5C).

**Table 2:** Potential keystone taxa in the AOA and AOB networks based on treatments without (OM−) and with (OM+) organic material amendments.

Network	Module	Role	Degree	Abundance (%)	Taxa	*Z* ^a^	*P* ^a^	AOA richness^b^	AOB richness^b^	PNA_total_^b^
OM−	Module II	Module hub	24	4.95	*Nitrosospira* cluster 12	2.56	0.12	0.33	−0.57^*^	0.48^*^
OM+	Module I	Module hub	27	0.18	*Nitrososphaera* cluster 9	3.03	0.21	0.74^**^	0.54^*^	0.73^**^
	Module I	Module hub	25	2.27	*Nitrososphaera* cluster 9	2.61	0.17	0.81^**^	0.73^**^	0.84^**^
	Module I	Module hub	22	0.01	*Nitrosospira* cluster 10	2.52	0.10	0.65^**^	0.52^*^	0.56^*^

a *The topological role of each node is determined according to the within-module connectivity *Z* and the among-module connectivity *P**.

b *PNA_total_, the sum of potential nitrification activity of AOA and AOB. The AOA and AOB richness are represented by Chao1 richness*.

** *p* *< 0.01;*

* *p* < 0.05.

### Predictors of Nitrification, Plant Productivity, and N Use Efficiency

Correlation analysis and random forest modeling were used to identify potential predictors of PNA_total_, plant productivity, and NUE, including soil properties, and the AOA and AOB populations. Correlation analysis revealed that PNA_total_, straw biomass, grain yield, and NUE were significantly associated with SOC, TN, NH_4_^+^-N, as well as with the abundance, Chao1 richness, and network (module eigengenes) of AOA and AOB populations (Supplementary Figure S3; *p* < 0.05). Random forest modeling indicated that TN (6.2–8.1%, *p* < 0.01), NH_4_^+^-N (5.6–7.5%, *p* < 0.01), and SOC (4.9–7.6%, *p* < 0.05) were the three major predictors of PNA_total_, straw biomass, grain yield, and NUE (Supplementary Figure S3). Besides, the abundance (4.9–7.9%, *p* < 0.05), Chao1 richness (4.9–5.9%, *p* < 0.05), composition (4.0–4.5%, *p* < 0.05), and network (4.1–4.6%, *p* < 0.05) of AOA and AOB populations contributed significantly to the variations in PNA_total_, straw biomass, grain yield, and NUE (Supplementary Figure S3).

**Figure 6:**
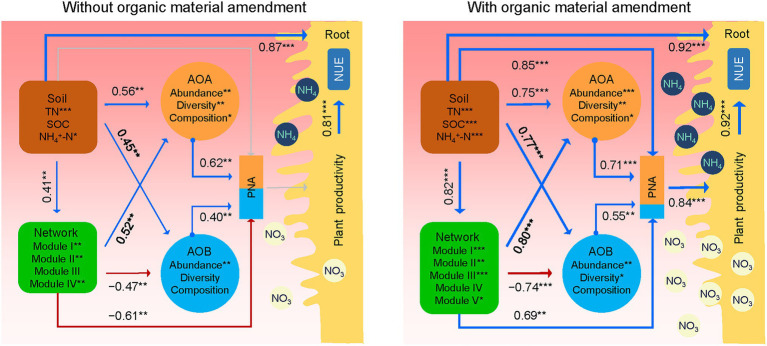
Schematic figure displaying the effects of soil properties, AOA and AOB populations on PNA_total_, plant productivity, and nitrogen use efficiency (NUE) based on structural equation modeling analysis of treatments without and with organic material amendments. The latent variables (soil properties, AOA and AOB, and network) inside the boxes were used to integrate the effects of multiple conceptually related variables into a single-composite effect. Soil properties include soil organic carbon (SOC), total nitrogen (TN), and ammonia nitrogen (NH_4_^+^-N). The AOA and AOB are represented by the *amoA* gene abundance, diversity (Chao1 richness), and composition (PCoA1). The co-occurrence networks are represented by module eigengenes. PNA is the sum of AOA and AOB potential nitrification activity, and the contributions of AOA and AOB to PNA were colored in orange and blue, respectively. Plant productivity is the sum of grain yield and straw biomass. Blue and red lines indicate positive and negative effects, respectively. The width of arrows indicates the strength of significant standardized path coefficients. Paths with non-significant coefficients are displayed as gray lines. ^***^p < 0.001; ^**^p < 0.01; ^*^p < 0.05.

Structural equation modeling further linked the potential influences of soil properties and AOA and AOB populations on PNA_total_, plant productivity, and NUE under treatments without and with organic material amendments (Figure 6). Overall, soil properties showed significant positive relationships with the AOA and AOB populations, the AOA-AOB network (module eigengenes), and plant productivity. The AOA-AOB network exhibited significantly negative associations with the AOB population, but positive associations with the AOA population. The AOA and AOB populations were positively related to PNA_total_ under treatments without and with organic material amendments. However, the AOA-AOB network was negatively correlated with PNA_total_ under treatments without organic material amendments but positively correlated with PNA_total_ under treatments with organic material amendments. This result pointed to the fact that the AOA population might exert a higher contribution to PNA_total_ than the AOB population, particularly with organic material amendments.

## Discussion

### Organic Material Amendments Affect the Structure of AOA and AOB Populations

Our results provided evidence that organic material amendments could significantly improve soil fertility, in particular, by increasing SOC, TN, and NH_4_^+^-N. Straw contains large amounts of nutrient elements, such as C and N, and its decomposition leads to the transformation of important molecules and available nutrients in agricultural soils (Wang et al., 2018). On a long-term scale, the decomposition of lignin, cellulose, and hemicellulose from straw will gradually facilitate the formation of stable SOC, and straw biochar can also be formed as a recalcitrant SOC, thus collectively accounting for SOC pool (Keith et al., 2015). Compared to the NS and NB treatments, the NSM treatment significantly promoted the organic N mineralization of straw material and increased the contents of NH_4_^+^-N and TN. The combined application of straw and manure decreases the C/N ratio of straw and effectively accelerates the decomposition affecting soil nutrient turnover rates (Qiao et al., 2015; Wang et al., 2021).

The changes in the composition of AOA and AOB populations can be used to investigate the potential impacts on soil nitrification. We found that organic material amendments significantly enhanced the abundance and diversity of AOA and AOB populations, and caused the variations in their respective population structures. The abundance and diversity of AOA and AOB populations increased with the higher contents of SOC, TN, and NH_4_^+^-N. Notably, the abundance of AOA was higher than AOB, most likely due to their better adaptation at a relatively low pH (4.42–4.75) of acidic soils (Teutscherova et al., 2017). Our study revealed that AOA *Nitrosotalea* cluster 1.1 and AOB *Nitrosospira* clusters 12 and 3b were enriched under CK and N treatments. *Nitrosotalea* cluster 1.1 and *Nitrosospira* clusters 12 and 3b are found to be dominate in acid soil, and their growth and nitrification activity are high at low pH (Chen et al., 2011; Gubry-Rangin et al., 2011). Organic material amendments display an overall higher nutrient availability that supports the larger abundance of AOA *Nitrososphaera* clusters 2 and 9, AOB *Nitrosospira* clusters 3a and 10 (Verhamme et al., 2011; Cong et al., 2020). Worth mentioning, both AOA and AOB are known to be highly diverse functional groups and the dominant phylotypes in the AOA (*Nitrososphaera* cluster 9) and AOB populations (*Nitrosospira* clusters 3a and 10) have often been documented to be functionally active in agricultural soils (Pester et al., 2012; Jiang et al., 2014; Ouyang et al., 2016).

There is growing evidence that soil pH and NH_3_ concentration have differential effects on the growth of AOA and AOB populations, indicating that AOA and AOB occupy separate ecological niches (French et al., 2021; Jung et al., 2022). Since these acidophilic AOA should be highly adapted to low pH value and NH_3_ concentration (Verhamme et al., 2011), more potential collaborative relationships between AOA population may be linked to nitrification activity in the OM− network. Compared with CK and N treatments (OM−), the abundance of AOA and AOB measured by the copy number of *amoA* gene was significantly increased under NS, NSM, and NB treatments (OM+). Co-occurrence network analysis revealed a higher percentage of negative correlations and connectivity in the OM+, potentially suggesting higher taxa competition that promoted network robustness. In particular, three potential keystone taxa (*Nitrososphaera* cluster 9 and *Nitrosospira* cluster 10) displayed negative associations with other connected OTUs, thus exhibiting more linkages between AOA and AOB in the negative relevance direction. This higher number of negative correlations between AOA and AOB in the OM+ likely reflect the competition for substrate, that is, NH_3_ (Hibbing et al., 2010; French et al., 2021). The concentration of NH_3_ present in a particular environment can change by orders of magnitude based on soil pH (Groeneweg et al., 1994; Jung et al., 2022). Based on these findings on cell kinetic properties, NH_3_ affinity is proposed as a determinant for habitat selection and niche differentiation between AOA and AOB populations (Schleper, 2010). AOA has a much higher affinity for NH_3_ than AOB in various extreme environments, indicating that their different survival or lifestyle strategies (Martens-Habbena et al., 2009). In contrast, the AOB population is favored at relatively high concentrations of NH_3_, contributing to strong competition for NH_3_ between AOA and AOB (Prosser and Nicol, 2012). Theoretically, high competition reinforced by keystone taxa exerts a profound contribution to maintaining the microbial diversity and functioning (Zhao et al., 2016). We speculated that high AOA and AOB diversity were sustained *via* the recruitment of particular keystone taxa from the indigenous species pool under organic material amendments. The potential keystone taxa in the networks are likely to explain a large proportion of the variation in the entire microbial community composition (Jiang et al., 2017; Herren and McMahon, 2018). Last, ecological theory suggests that resource competition reinforced by keystone taxa can promote microbial diversity, community structural integrity, and network stability, in particular when taxa share similar resource requirements and niche preferences (Banerjee et al., 2018; Chen et al., 2019).

### Changes in AOA and AOB Populations Affect PNA, Plant Productivity, and NUE

Our results revealed that the increases in ammonia oxidizers (AOA and AOB) abundance and diversity were significantly correlated with PNA_total_, plant productivity, and NUE under treatments with organic material amendments. We found that PNA_AOA_ was significantly higher than PNA_AOB_, except under the NB treatment. Values of PNA_total_ were most strongly correlated with the abundance of AOA than that of AOB across treatments. These results corroborate previous observations that AOA is more important than AOB for ammonia oxidation in acidic soils (Stopnisek et al., 2010; Yao et al., 2011). This difference in niche preferences between AOA and AOB taxa occurs mostly due to distinct cell affinities for NH_3_ (Martens-Habbena et al., 2009; Rütting et al., 2021). Besides, it is worth noticing that the higher abundance of the population should be cautioned interpreted with respect to their activities (Nicol et al., 2008; Jiang et al., 2014). Last, the higher abundance of AOB under the NB treatment can be explained by the fact that biochar can ameliorate soil acidity and improve fertility, which collectively would have favored AOB over AOA. Accordingly, the AOA and AOB populations were proposed to be responsible for approximately equal nitrification potential under successive biochar treatment in acidic soils.

We further found that potential competitive interactions mediated by potential keystone taxa in the AOA-AOB networks facilitated positive diversity-functioning relationships. These keystone taxa often serve as structural units within the network and are expected to exert significant contributions to PNA and N dynamics irrespective of their abundance (Lynch and Neufeld, 2015). The functional traits of keystone taxa have frequently been proposed to be of particular ecological importance in determining the relationship between taxa richness and nutrient dynamics (Banerjee et al., 2018; Yang et al., 2021). A meta-analysis based on microbial diversity manipulation indicated that 80% of the studies support significantly positive relationships between species richness and microbial-derived ecosystem functions (Saleem et al., 2019). We corroborated this notion by showing that greater soil microbiome diversity—in this case, mediated by keystone taxa—stimulated community performance acting on nitrogen cycling, and increased plant productivity and NUE. The high microbiome diversity sustained *via* the potential competitive interactions induced by keystone taxa may contribute to the eventual promotion of soil multifunctioning in the natural field systems (Chen et al., 2019; Yang et al., 2021). As such, our results suggested that the intensely negative correlations in the networks of AOA and AOB associations could accelerate the community performance of nitrification at high levels of diversity. However, it should be noted that the positive effects of keystone taxa on PNA were basically based on statistical correlations, because of the fact that most AOA and AOB in soils are uncultured (Li et al., 2018). We still lack direct evidence for the “true” impact of statistically identified keystone taxa on the AOA and AOB populations. Therefore, stable isotope tracking is required to further disentangle the underlying mechanisms of keystone taxa regulating ammonia oxidizers community and soil nitrification.

## Conclusion

Our study revealed that long-term organic material amendments could significantly increase soil fertility and lead to variations in the abundance, diversity, and population structures of AOA and AOB. Co-occurrence network analysis suggested that organic material amendments enhanced negative correlations between ammonia oxidizers, largely mediated by potential keystone taxa. These keystone taxa were interpreted as important for the overall diversity of AOA and AOB populations and directly associated with rates of PNA. Besides, this study corroborates the literature by showing the greater importance of AOA over AOB for nitrification in acidic soils. These results are directly linked with PNA, plant productivity, and NUE, likely due to the higher abundance and diversity of AOA and AOB populations under organic material amendments. Taken together, this study integrates the ecology of ammonia oxidizers with experimental-based evidence to provide insights into keystone taxa mediating potential interactions and nitrification under organic material amendments. Our study holds great promise for crop productivity and NUE through the application of keystone taxa in targeted microbiome manipulation.

## Data Availability Statement

The datasets presented in this study can be found in online repositories. The names of the repository/repositories and accession number(s) can be found at: https://www.ncbi.nlm.nih.gov/genbank/, PRJNA793074, https://www.ncbi.nlm.nih.gov/genbank/, PRJNA793076.

## Author Contributions

YJ and LT designed all the experiments. JZ, YJ, QX, and FD-A wrote the manuscript. JZ, LL, PK, JX, and GZ were responsible for performing the field and lab experiments. All authors analyzed all data, discussed the results, critically reviewed the manuscript, and approved its publication. All authors read and approved the final manuscript.

## Funding

This research was financially supported by National Science Fund for Excellent Young Scholars of China (41922048), National Natural Science Foundation of China (42177298 and 41771297), Youth Innovation Promotion Association of CAS (Y2021084), State Key Laboratory Program of China (Y21200004), and China Postdoctoral Science Foundation (2021M690155).

## Conflict of Interest

The authors declare that the research was conducted in the absence of any commercial or financial relationships that could be construed as a potential conflict of interest.

The handling editor declared a shared affiliation with the authors JZ, LL, PK, JX, GZ, and YJ at the time of the review.

## Publisher’s Note

All claims expressed in this article are solely those of the authors and do not necessarily represent those of their affiliated organizations, or those of the publisher, the editors and the reviewers. Any product that may be evaluated in this article, or claim that may be made by its manufacturer, is not guaranteed or endorsed by the publisher.
