# Supplementary material for: Dynamic Responses of Ammonia-Oxidizing Archaea and Bacteria Populations to Organic Material Amendments Affect Soil Nitrification and Nitrogen Use Efficiency
Source: Front Microbiol. 2022 May 12;13:911799. doi: 10.3389/fmicb.2022.911799 (PMC9135446; doi:10.3389/fmicb.2022.911799)
Supplement: Supplementary file 2 [file Table_1.docx]

**Supplementary Materials for**

**Dynamic responses of ammonia-oxidizing archaea and bacteria populations to organic material amendments affect soil nitrification and plant productivity**

Jie Zheng^1,2†^, Liang Tao^3†^, Francisco Dini-Andreote^4^, Lu Luan^4^, Peijun Kong^1,2^, Jingrong Xue^1^, Guofan Zhu^1^, Qinsong Xu^5^, Yuji Jiang^1,^ *

^1^ State Key Laboratory of Soil and Sustainable Agriculture, Institute of Soil Science, Chinese Academy of Sciences, Nanjing, China.

^2^ University of Chinese Academy of Sciences, Beijing, China.

^3^ Guangdong Key Laboratory of Integrated Agroenvironmental Pollution Control and Management, Institute of Eco-environmental and Soil Sciences, Guangdong Academy of Sciences, Guangzhou, China.

^4^ Department of Plant Science & Huck Institutes of the Life Sciences, The Pennsylvania State University, University Park, PA, USA.

^5^ College of Life Science, Nanjing Normal University, Nanjing, China.

^†^ These authors contributed equally to this work.

*** Corresponding authors:**

Yuji Jiang yjjiang@issas.ac.cn

**This file includes:**

Figures S1 to S3

Tables S1


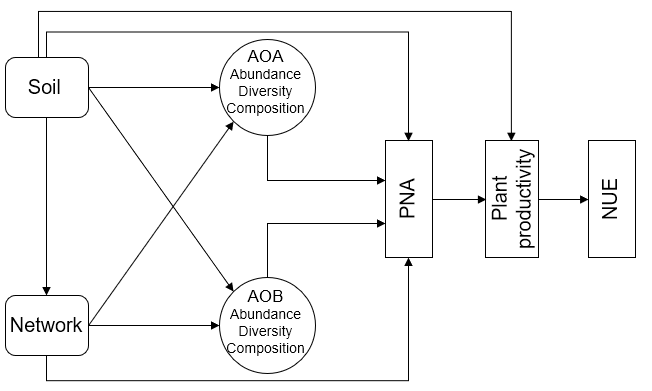


**Figure S1.** The framework and rationale of the priori structural equation modelling (SEM). SEM is performed to assess potential direct and indirect effects of soil properties and AOA and AOB populations on potential nitrification activity (PNA), plant productivity, and nitrogen use efficiency (NUE).


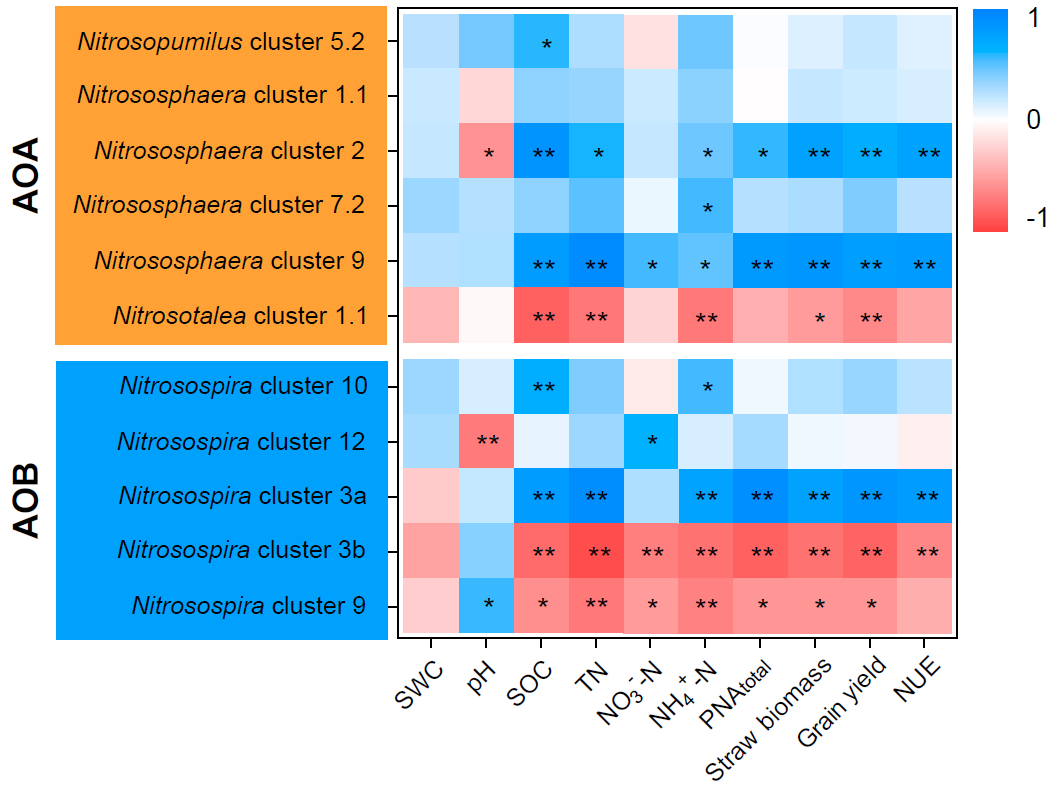


**Figure S2.** Correlation coefficients between the relative abundance of AOA and AOB taxa and soil properties, PNA_total_, plant productivity (grain yield and straw biomass), and NUE. SWC, soil water content; SOC, soil organic carbon; TN, total nitrogen; NO_3_^−^-N, nitrate nitrogen; NH_4_^+^-N, ammonia nitrogen; PNA_total_, the sum of potential nitrification activity of AOA and AOB populations. ^**^*P* < 0.01; ^*^*P* < 0.05.


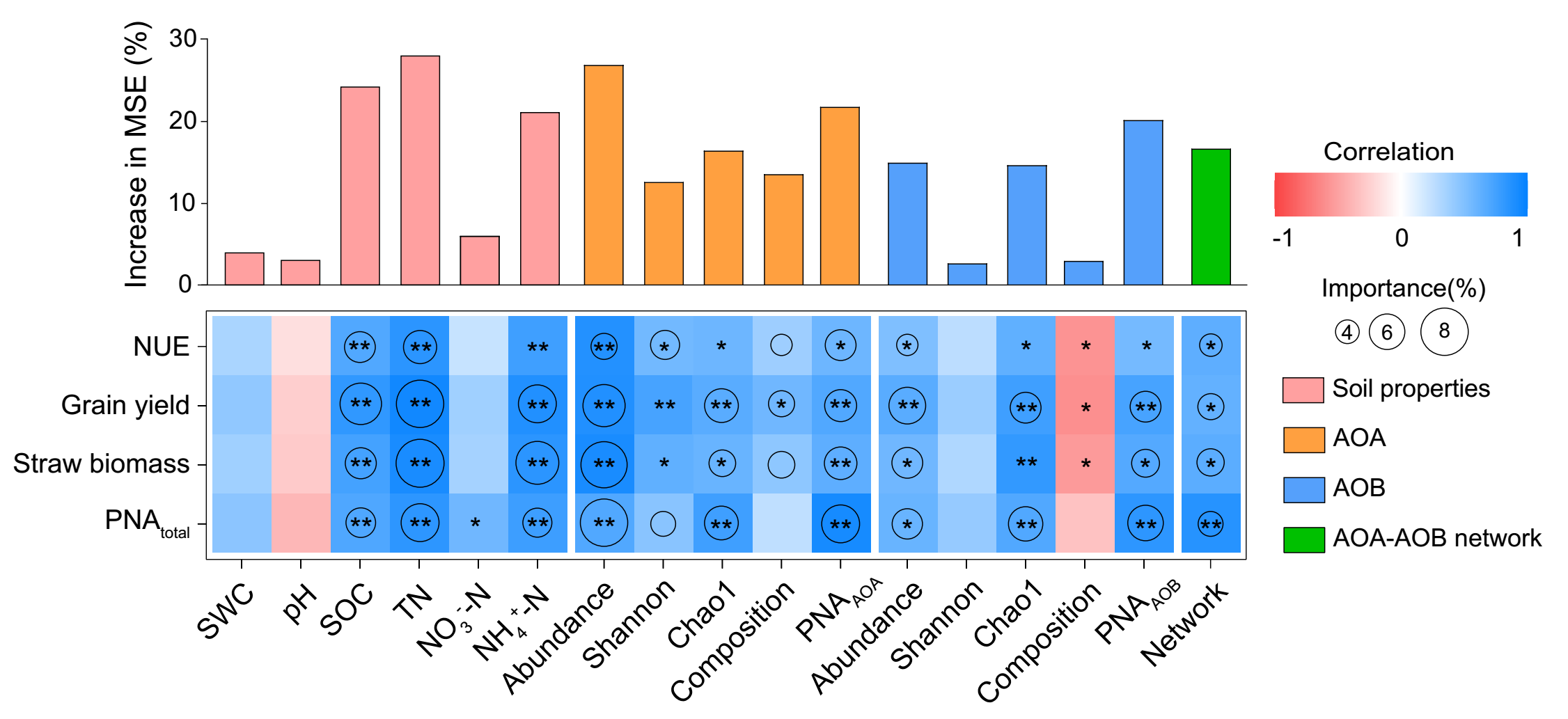


**Figure S3.** Contributions of soil properties, AOA and AOB, and co-occurrence network to PNA_total_, plant productivity (grain yield and straw biomass), and nitrogen use efficiency (NUE) were evaluated by correlational and random forest model analyses. Circle sizes represent the variables’ importance (% of increased mean square error, calculated via the random forest model, MSE). The composition of AOA and AOB were indicated by the first principal coordinates (PCoA1). The network was represented by the first principal coordinates of module eigengenes (PCoA1). Colors represent Spearman’s correlation coefficients. SOC, soil organic carbon; TN, total nitrogen; NO_3_^−^-N, nitrate nitrogen; NH_4_^+^-N, ammonia nitrogen; PNA_AOA_, the potential nitrification activity of AOA; PNA_AOB_, the potential nitrification activity of AOB; PNA_total_, the sum of potential nitrification activity of AOA and AOB. ^**^*P* < 0.01; ^*^*P* < 0.05.

**Table S1** Topological properties of AOA and AOB co-occurrence networks based on treatments without (OM−) and with (OM+) organic material amendments.

| Network metrics | OM− | OM+ |
| --- | --- | --- |
| Number of nodes | 57 | 85 |
| Number of edges | 277 | 355 |
| Number of positive correlations | 149 | 128 |
| Number of negative correlations | 128 | 227 |
| Percentage of negative correlations (PNC, %) | 46.2 | 63.9 |
| Average path length (APL) | 2.172 | 2.648 |
| Graph density | 0.174 | 0.199 |
| Network diameter | 5 | 8 |
| Average clustering coefficient (*avg*CC) | 0.29 | 0.298 |
| Average degree (*avg*K) | 9.72 | 8.35 |
| Number of modules | 4 | 5 |
| Modularity | 0.616 | 0.654 |
